# Implementing a Birth Dose of Hepatitis B Vaccine in Africa: Findings from Assessments in 5 Countries

**Published:** 2018-07-02

**Authors:** Edna Moturi, Carole Tevi-Benissan, José E. Hagan, Stephanie Shendale, David Mayenga, Daniel Murokora, Minal Patel, Karen Hennessey, Richard Mihigo

**Affiliations:** 1 World Health Organization Regional Office for Africa, Brazzaville, Republic of Congo; 2 Global Immunization Division, Centers for Disease Control and Prevention, Atlanta, GA, USA; 3 World Health Organization, Expanded Programme on Immunization, Geneva, Switzerland

**Keywords:** Hepatitis, Birth Dose, Africa, WHO

## Abstract

**Introduction:**

Few African countries have introduced a birth dose of hepatitis B vaccine (HepB-BD) despite a World Health Organization (WHO) recommendation. HepB-BD given within 24 hours of birth, followed by at least two subsequent doses, is 90% effective in preventing perinatal transmission of hepatitis B virus. This article describes findings from assessments conducted to document the knowledge, attitudes, and practices surrounding HepB-BD implementation among healthcare workers in five African countries.

**Methods:**

Between August 2015 and November 2016, a series of knowledge, attitude and practices assessments were conducted in a convenience sample of public and private health facilities in Botswana, the Gambia, Namibia, Nigeria, and São Tomé and Príncipe (STP). Data were collected from immunization and maternity staff through interviewer-administered questionnaires focusing on HepB-BD vaccination knowledge, practices and barriers, including those related to home births. HepB-BD coverage was calculated for each visited facility.

**Results:**

A total of 78 health facilities were visited: STP 5 (6%), Nigeria 23 (29%), Gambia 9 (12%), Botswana 16 (21%), and Namibia 25 (32%). Facilities in the Gambia attained high total coverage of 84% (range: 60–100%) but low timely estimates 7% (16–28%) with the median days to receiving HepB-BD of 11 days (IQR: 6–16 days). Nigeria had low total (23% [range: 12–40%]), and timely (13% [range: 2–21%]) HepB-BD estimates. Facilities in Botswana had high total (94% [range: 80—100%]), and timely (74% [range: 57—88%]) HepB-BD coverage. Coverage rates were not calculated for STP because the maternal Hepatitis B virus (HBV) status was not recorded in the delivery registers. The study in Namibia did not include a coverage assessment component. Barriers to timely HepB-BD included absence of standard operating procedures delineating staff responsible for HepB-BD, not integrating HepB-BD into essential newborn packages, administering HepB-BD at the point of maternal discharge from facilities, lack of daily vaccination services, sub-optimal staff knowledge about HepB-BD contraindications and age-limits, lack of outreach programs to reach babies born outside facilities, and reporting tools that did not allow for recording the timeliness of HepB-BD doses.

**Discussion:**

These assessments demonstrate how staff perceptions and lack of outreach programs to reach babies born outside health facilities with essential services are barriers for implementing timely delivery of HepB-BD vaccine. Addressing these challenges may accelerate HepB-BD implementation in Africa.

## Introduction

Hepatitis B virus (HBV) is a major cause of cirrhosis, liver cancer, and end-stage liver disease^1^,^2^. HBV is highly infectious and is transmitted by exposure to infected blood and other body fluids such as semen and vaginal fluid3. Most morbidity from HBV is due to chronic infection. An estimated 240 million people are infected with HBV worldwide, and more than 686,000 deaths are attributable to chronic HBV complications annually^2^,^4^,^5^. The likelihood of developing chronic HBV is highest if infection occurs at time of birth, and approximately 70–90% of persons infected perinatally progress to develop chronic HBV infection^3^,^6^,^7^.

With only 16% of the world’s population, countries in the World Health Organization’s (WHO) African Region are disproportionally affected, and have the highest endemicity worldwide (8.83%, defined as having a chronic hepatitis B surface antigen (HBsAg) population prevalence of ≥8%^8^,^9^. Despite the availability of an effective vaccine, approximately 5–10% of the adult population is chronically infected and chronic HBV is a leading cause of death for young adults in the region, most of who are unaware of their infection until the disease has progressed to late stages^2^,^10^,^11^. In highly endemic settings, HBV is mainly transmitted perinatally from mother-to-child at the time of birth, and through horizontal transmission during early childhood^12^. WHO recommends universal vaccination with a monovalent dose of hepatitis B (HepB) vaccine (HepB-BD) given within 24 hours of birth, followed by at least two subsequent doses^3^. Vaccination has been shown to dramatically reduce the post-vaccination prevalence of HBsAg carriage in children less than 5 years^13^. Timely HepB-BD (given within 24 hours after birth), followed by at least two doses is 90% effective at preventing perinatal transmission^3^,^7^.

All African countries have incorporated HepB vaccine in their Expanded Programme for Immunization (EPI) schedules. The vaccine is usually given as three doses of the pentavalent vaccine (diphtheria, tetanus, pertussis, Haemophilus influenza type B, and HepB) at 6, 10, and 14 weeks of age^11^. Regional coverage for the third dose of HepB (HepB3) has increased from 5% in 2001 to 76% in 2015; however, this is below the global coverage of 84%^14^. Individual country estimates range from 16% in Equatorial Guinea to 98% in Rwanda, Seychelles, Swaziland and the United Republic of Tanzania, with significant variation at district level. African countries have been slow to implement a 2009 WHO recommendation to introduce HepB-BD, and so far only 23% of children in the region are benefiting from the vaccine^15^. These children reside in 11 countries that have introduced HepB-BD (Algeria, Angola, Botswana, Cape Verde, the Gambia, Mauritania, Mauritius, Namibia, Nigeria, São Tomé and Príncipe [STP], and Senegal)^14^. In November 2014, during the Sixty-fourth session of the Regional Committee, the African Region adopted a resolution to reduce chronic HBV infection to less than 2% in children under 5 years of age, and to introduce HepB-BD in at least 25 countries by the end of 2020^16^.

Timely HepB-BD implementation poses programmatic challenges which should be considered prior to its introduction^17^,^18^. Even the Gambia which was one of the first countries to introduce HepB-BD, is reaching less than 3% of newborns with timely HepB-BD^19^,^20^. Multiple factors have been documented to affect implementation of HepB-BD^21^–^23^. Of these, human resource factors e.g., staff shortages, lack of training opportunities, poor attitude and gaps in knowledge among healthcare staff are frequently associated with poor uptake of immunization programs in Africa^24^,^25^. The effect of healthcare workers (HCWs) is especially important when it comes to implementing HepB-BD given the timing recommendations of the vaccine.

With the recent resolution to increase timely HepB-BD coverage in the region, is important to first understand the experiences from the few countries that have already introduced HepB-BD. This will help guide other countries who are in the process of operationalizing HepB-BD or seeking to strengthen existing programmes. This paper presents findings from assessments conducted to determine the level of knowledge, attitudes, and practices surrounding HepB-BD administration among HCWs in five African countries.

## Methods

**Figure 1:**
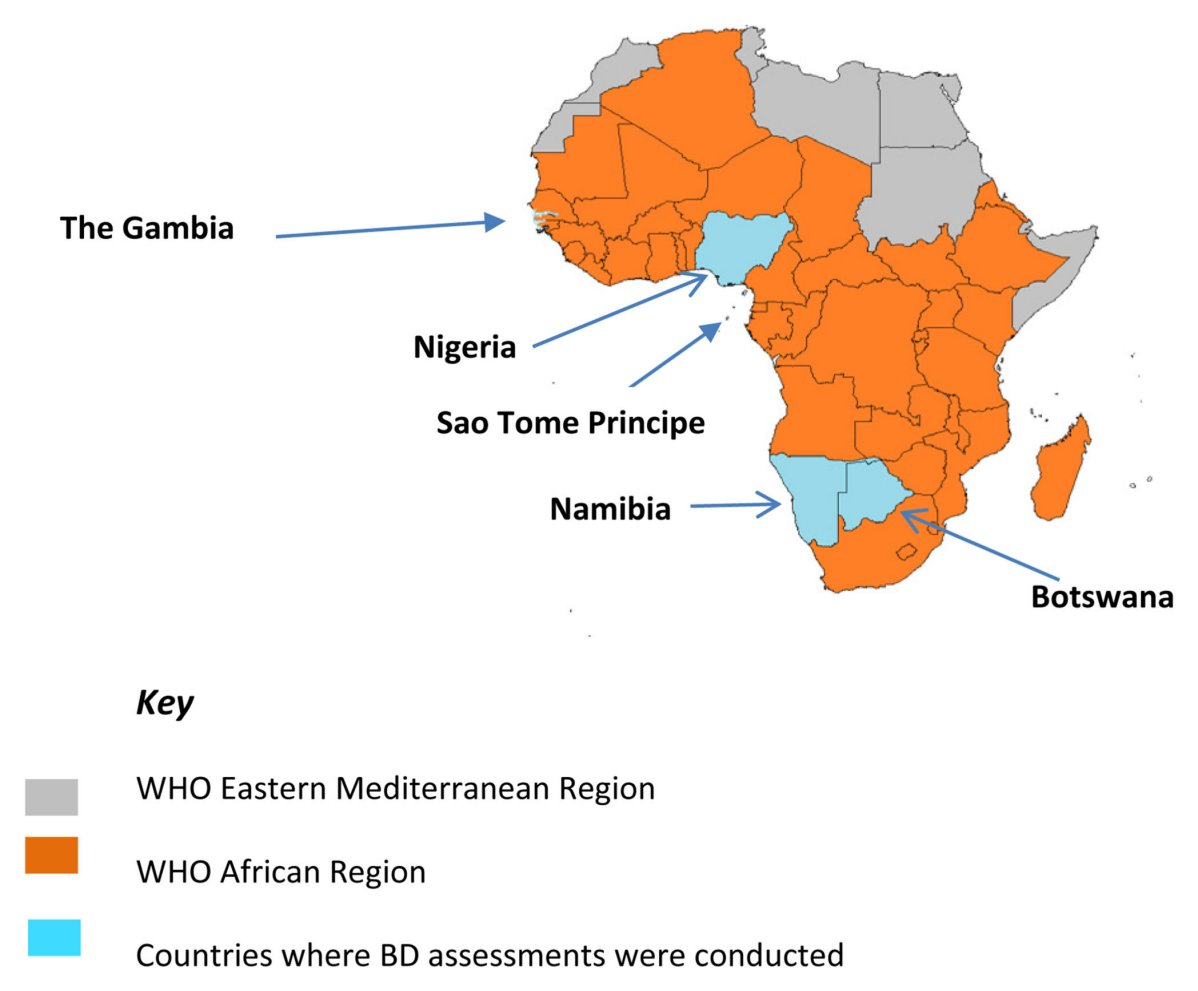
Map showing the 5 countries where BD assessments were conducted

Between August 2015 and November 2016, cross-sectional studies were carried out in select administrative regions in Botswana, the Gambia, Namibia, Nigeria and STP (Figure 1). The regions were selected based on the previous years’ reported HepB-BD coverage and identification as a priority area by EPI staff based on low immunization system performance as indicated by coverage with three doses of DTP (DTP3) vaccine. Within each region, at least two provinces were selected based on HepB-BD coverage and operational feasibility.

Attempts were made to include at least one high and one low performing province. Provinces with <10% coverage were excluded a priori. In each province, convenience sampling was done to identify a sample of 6 to 8 health facilities that provided delivery services. The main national referral hospitals in each country were selected. Private facilities were deliberately selected in cities such as Lagos with a heavy private sector presence.

At national level, health policies and programme documents including previous EPI reviews and recent Gavi progress reports were reviewed to obtain basic programmatic data. A review of WHO and the United Nations Children’s Fund (UNICEF) immunization coverage estimates which are based on data reported by countries through the Joint Reporting Form (JRF) was conducted. Interviews were conducted with Ministry of Health (MoH) officials regarding national HepB-BD and Maternal and Child Health (MCH) policies, training, supervision and vaccine management.

Evaluators drawn from MoH and WHO were trained to collect data using common assessment questionnaires which were developed during a February 2015 HepB-BD consultative workshop which was held at the WHO Regional Office for Africa (AFRO) in Brazzaville, Republic of Congo. The questionnaires contained structured and unstructured questions surrounding HepB-BD implementation. At each visited health facility, EPI and maternity supervisors were interviewed about HepB-BD vaccination policy, practices, knowledge and barriers, including those related to home births. They were questioned about any previous training in basic emergency obstetric and neonatal care (BEmONC), and whether the trainings endorsed the provision of timely HepB-BD. Evaluators observed vaccine handling and storage at each health facility. For each facility, data was collected on the number of births that had taken place in the year preceding the assessment, and the number of infants who had received a dose of HepB-BD. Finally, a record-based coverage assessment was conducted for each facility to calculate two estimates: (1) total HepB-BD (doses administered any time after birth), and timely HepB-BD (doses given on the day of birth or the day after). A random sample of recent births was identified from the maternity register and later searched for in the immunization registry. The date of the vaccine dose was compared against the date of birth to determine the timing of HepB-BD administration. A modified protocol was employed in Namibia as the HepB-BD assessment was embedded in a larger comprehensive EPI and surveillance review and did not include a record-based coverage assessment component.

Data were entered, cleaned and analyzed using a Microsoft Excel database^26^. Descriptive statistics like frequencies and proportions were used to summarize the data. The assessments were funded by WHO and were exempted from ethical approval for human subjects from institutional review boards.

## Results

**Figure 2:**
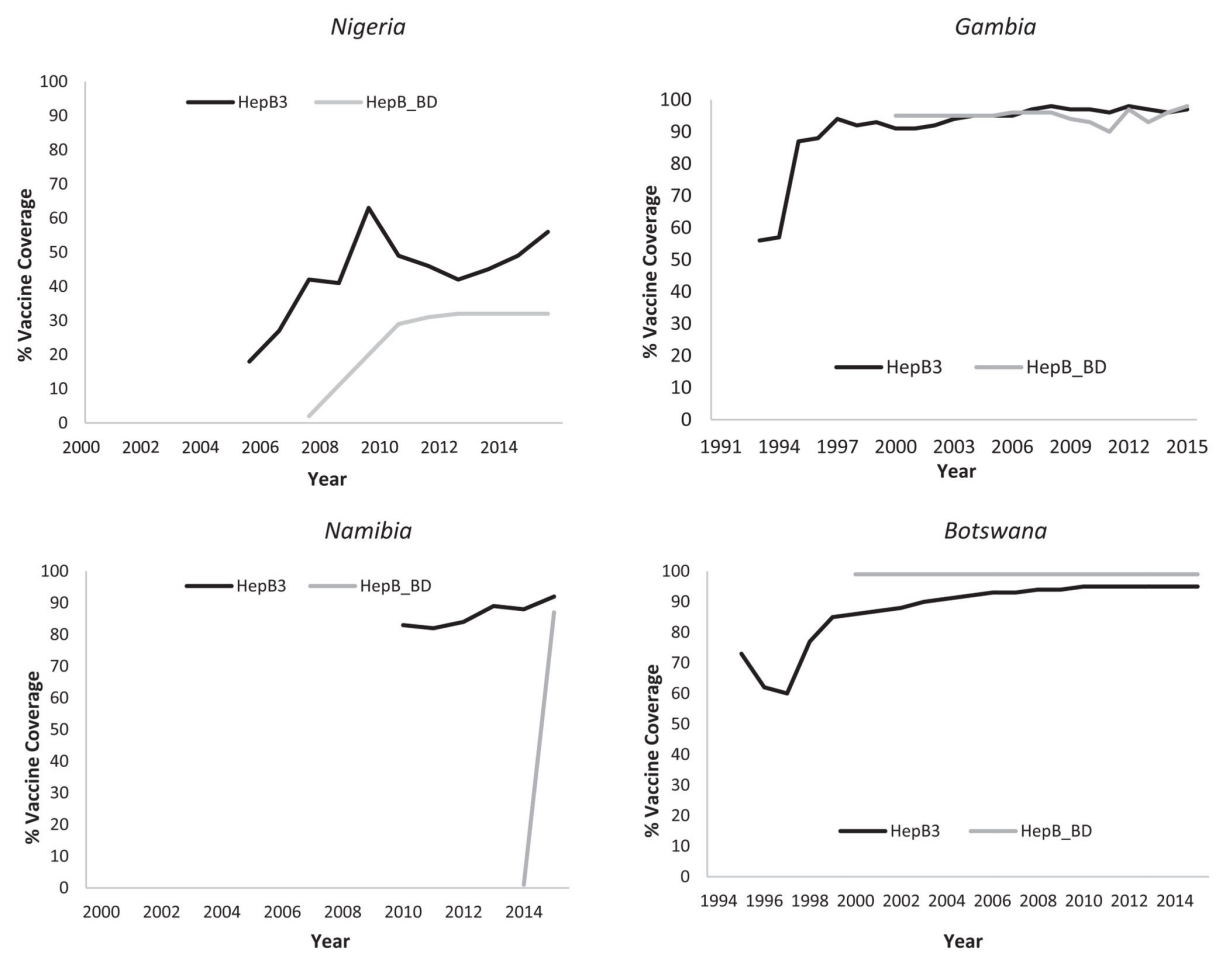
Childhood Hepatitis B vaccination coverage in the countries with birth dose assessments

A total of 78 health facilities were visited during the assessments: STP 5 (6%), Nigeria 23 (29%), Gambia 9 (12%), Botswana 16 (21%), and Namibia 25 (32%) (Figure 1). The health facilities were a combination of public hospitals and health centers and private health facilities. All 78 facilities provided hepatitis B vaccination services (Table 1). The total number of deliveries per year in the selected health facilities ranged from 167 (range: 19–6,000) in Botswana to 1719 (range: 500–6,000) in the Gambia. All health facilities in STP were keeping mothers in the post-natal ward for at least 24 hours after delivery, as compared to facilities in Nigeria (43%), the Gambia (33%), and (13%). (Figure 2)

A total of 1,196 records were evaluated in the three countries where capture-recapture sampling was conducted: Botswana (400), Nigeria (571), and the Gambia (225). HepB-BD coverage calculations were not possible in STP because maternal HBsAg status was not documented in delivery registers. High total HepB-BD estimates were attained in the Gambia 84% (range: 60–100%), though the timely estimate was significantly lower 7% (range: 16–28%). Only 9% of infants who received HepB-BD in the Gambia were vaccinated within 24 hours of birth. The median days to receiving HepB-BD was 11 days (IQR: 6–16 days), and majority (42%) were vaccinated between 8 to 14 days. Nigeria had low total 23% (range: 12–40%), and timely HepB-BD coverage 13% (range: 2–21%). Botswana had high total HepB-BD coverage estimates (94% [range: 80—100%]), with the highest found in facilities in Kwaneng West province (98%), and the lowest in Gaborone (89%). However, Kwaneng West facilities had the lowest timely HepB-BD estimates (62%). Private hospitals had the highest total coverage (99%), and the National referral hospital followed closely at 80%. Overall, timely HepB-BD coverage in Botswana was 74% (range: 57-88%).

## Overview of the National HepB-BD programmes

All five countries are highly endemic for HBV (Table 2). However, none of the 5 countries had conducted nationally representative seroprevalence assessments. With the exception of STP, all are countries have policies for universal vaccination with monovalent HepB-BD given soon after birth, and 3 doses of pentavalent vaccine. STP introduced HepB-BD in 2002 and is using a selective screening and targeted vaccination approach. Women are routinely screened for HBsAg during antenatal care (ANC) visits, and only babies of HBV-seropositive (HBV+) mothers receive HepB-BD. HBV+ mothers were counselled on importance of delivering in hospitals to ensure their newborns receive HepB-BD and other postnatal care (PNC) services. In 2015, STP reported high national coverage for HepB3 (96%) but low HepB-BD (3%)^14^. This low rate is thought to be due to denominator issues which did not allow for appropriate statistical analysis. However, coverage among infants born to HBV+ women is high (92%). Nigeria introduced HepB-BD in 2004 but the 2015 coverage estimates for both HepB-BD (32%) and HepB3 (56%) are low. The Gambia introduced HepB-BD in the 1990s and has consistently maintained high coverage rates for HepB-BD (98%) and HepB3 (97%). Botswana introduced HepB-BD in the 1990s and has maintained high (90%) national coverage rates. Namibia introduced HepB-BD in 2014 and in 2015 attained a high post-introduction coverage of 87%, and 92% coverage rate for HepB3.

HBV is a priority health issue in all five countries, and there is a strong political support to implement HepB-BD. All countries had national policies for the timing of HepB-BD administration, ranging from within 24 hours in Nigeria to up to 2 weeks after birth in Namibia (Table 3). A change in the vaccination policy in Nigeria in February 2015 restricted HepB-BD administration to only within 24 hours of birth and EPI tools were revised accordingly. National EPI reporting and recording tools in all 5 countries had designated columns for HepB-BD, though some health facilities were still using outdated versions which did not have these columns. Healthcare facilities routinely documented HepB-BD doses on EPI tally sheets and reporting forms, but none had modified maternity registers to include dedicated columns for HepB-BD. MCH and EPI policies were well integrated in STP which facilitated collaboration on HepB-BD implementation. The following gaps were observed: (1) none of the 5 countries had national viral hepatitis action plans, (2) none had conducted a nationally representative HBV serosurvey, (3) only Nigeria had designated the Human Immunodeficiency Virus (HIV) program to spearheaded the development of a draft national policy for viral hepatitis, though this was done without EPI involvement, (4) none had national clinical guidelines for managing viral hepatitis, (5) only Botswana recorded HepB-BD data in both EPI and delivery registers, (6) only the Gambia had adapted EPI recording/reporting tools to capture both timely and total HepB-BD doses (tools revised in 2015) (7) none had integrated HepB-BD in the BEmONC package, and (8) none had formal outreach programs to vaccinate babies born outside health facilities.

## Perspectives at the Service Delivery Level

### Knowledge, practices and missed opportunities to timely HepB-BD coverage

Staff frequently reported a lack of specific training for HepB-BD ranging from to 56% in the Gambia to 88% in Botswana. Despite this, knowledge regarding HBV was high among interviewed HCWs, and most healthcare workers (HCWs) in Botswana and STP knew that HepB-BD should be administered within 24 hours for maximum effectiveness. However, most interviewed staff had suboptimal knowledge regarding the age-limits and correct contraindications for HepB-BD. Additionally, HCWs in Nigeria were observed to have higher awareness for Bacillus Calmette-Guérin (BCG) than HepB-BD. Vaccination was a highly acceptable intervention in all five countries and no community refusal was observed. Vaccination services were offered daily in health facilities in STP and Botswana, including weekends while mothers remained hospitalized in maternity facilities. Vaccination often took place in the EPI clinic and was offered free of charge in all public facilities. However, private facilities charged small service fees. Private facilities in Nigeria and Namibia received their vaccine supply from the MoH in exchange for submitting monthly reports.

None of the staff interviewed in the 5 countries required senior authorization to administer HepB-BD. The Gambia, STP, and Botswana reported high antenatal care (ANC) attendance. Moreover, STP (91%) and Botswana (100%) reported high rates of facility delivery In STP, pregnant women who presented for delivery in health facilities were assessed for their HBsAg status. Those with no proof of ANC attendance, either ANC card or clinic records, were tested again in order to determine whether their infant was eligible for vaccination. False contraindications were commonly reported, including not vaccinating premature, very low birth-weight (<2kg), or ill but stable infants. HCWs in STP delayed initiating breastfeeding until HepB-BD administration for fear of transmission via breas tmilk.

### Administrative and documentation barriers to timely HepB-BD

With the exception of a few facilities in Namibia (36%) and Nigeria (26%), none of the assessed health facilities had written protocols for HepB-BD. STP’s policy of maternal screening and selective HepB-BD vaccination was unique among the evaluated countries and was identified as the principal barrier to achieving high HepB-BD coverage. With the exception of 2 (13%) facilities in Botswana, none reported vaccine stock-outs of over one month in the year preceding the evaluation. All countries implemented the multi-dose vial policy. The quality of cold chain was good in all facilities in STP, the Gambia and Botswana. However, 52% and 12% of facilities in Nigeria and Namibia respectively, lacked EPI-approved fridges. With the exception of one facility in Nigeria, no facilities had expired vaccines in the fridges. All facilities routinely documented HepB-BD on EPI tools, but only Botswana had revised their MCH tools to capture HepB-BD doses, while the Gambia had revised the EPI tools to capture both timely and total HepB-BD doses. District and national HepB-BD coverage calculations in all 5 countries did not specify the timing of HepB-BD vaccination. Few facilities analyzed immunization data and utilized findings for programmatic planning.

Major barriers to timely HepB-BD observed during the assessments included: (1) none of the 5 countries had outreach programs to vaccinate babies who were born outside health facilities, yet countries like Nigeria and the Gambia had high proportion of home births, (2) vaccinating babies at the time of discharge instead of at birth e.g., 50% of facilities in Botswana, (3) lack of awareness about appropriate age-limits among HCWs made them refuse to vaccinate eligible babies e.g., only 16% of HCWs in Namibia would administer HepB-BD if the baby was born at home and brought to health facilities even if within the 14 days, (4) cultural factors such as waiting until after the child’s naming day (around 7 days) before bringing them for PNC in the Gambia and Nigeria, and (5) lack of vaccination services during weekends and public holidays.

## Discussion

Findings from the HepB-BD assessments demonstrate the variability of birth dose implementation and the challenges countries face in immunizing babies with timely dose of HepB-BD. In 2015, the coverage for HepB-BD in the African region was only 10%, compared to the Global estimate of 39%14. Among the 11 countries implementing HepB-BD, coverage ranged from 19% in Angola to 99% in Algeria and Botswana. Facility estimates reported in the assessments correlated to the national reported estimates. Overall, visited facilities recorded lower timely HepB-BD estimates when compared to total rates. This could not be compared to national estimates because not all countries report timely HepB-BD coverage in the Joint Reporting Form (JRF), though revisions are underway.

### Barriers to increasing HepB-BD coverage in AFR – from assessments

Reaching babies within 24 hours of birth was a difficult problem in all five countries. Barriers to the timely administration of HepB-BD included weaknesses in national policies and lack of written guidelines to standardize HepB-BD implementation at health facilities. Evaluators did not find standard operating procedures (SOPs) at facilities to delineate when and where babies ought to be vaccinated, and which staff cadre was responsible for HepB-BD. As a result, many facilities were vaccinating babies at the point of hospital discharge instead of at birth. Vaccination services were often offered for half a day on weekdays and not on weekends or public holidays, and HepB-BD was not integrated into BEmONC packages in all countries.

Lack of training opportunities for HCWs was almost universally reported in all five countries. HepB-BD was often delayed or not given to infants because of inappropriate contraindication. Deficiency in knowledge about vaccine safety by HCWs has been linked to missed opportunities to vaccinate and low coverage rates^27^,^28^.

Previous assessments in the Gambia and the Philippines revealed the importance of increasing awareness and knowledge about HepB-BD vaccines among health workers to avoid false contraindication and missed opportunities to vaccinate^20^,^21^. The only contraindication to HepB-BD is anaphylactic reaction due to previous exposure. As such, all newborns are eligible for vaccination^3^. HCWs in STP withheld breastfeeding until HepB-BD vaccination because they falsely thought that breast milk could transmit HBV. Singling out HBV+ mothers during ANC and labor could lead to stigmatization and make women fearful of delivering in hospitals.

### Opportunities to improve HepB-BD coverage - from assessments

Governments in all five countries procured HepB-BD and provided it free of charge in public and private facilities. Advocacy by stakeholders including physician/nurses associations, the private sector, and hepatology associations in STP and Nigeria were integral in advocating for HepB-BD. Countries with a high proportion of institutional deliveries like Botswana (100%) and STP (91%) achieved high coverage in part due to ease of accessing the babies^29^. High HepB-BD estimates in delivery facilities with on-site vaccination clinics, and having both services in the same facility made it convenient for mothers to access vaccination services thereby increasing coverage.

Botswana, the Gambia and STP which attained high coverage estimates, have high ANC attendance rates which provide opportunities for educating mothers on HepB-BD and importance of delivering in facilities. Most facilities in STP were retaining mothers in the post-natal wards for at least 24 hours after delivery, which presents an opportunity to vaccinate newborns. These assessments demonstrate the positive impact that offering daily vaccination services, including weekends and public holidays have on increasing coverage. Facilities that vaccinated babies in the delivery wards at time of birth reported higher coverage than those vaccinating at the point of discharge. With the heavy workload that HCWs face, it is important to provide written guidelines, refresher trainings and supportive supervision to emphasize HepB-BD vaccination. One private hospital in Nigeria that had high coverage developed a checklist form for staff to fill out prior to discharging mothers from the labor ward. Such tools remind HCWs to administer HepB-BD and might improve HepB-BD coverage. Facilities in STP that stored HepB-BD in labor wards also had high coverage estimates. It was also observed that most facilities had high coverage rates of birth vaccines (BCG and first dose of polio vaccine [OPV]), which presents an opportunity to incorporate HepB-BD into essential newborn care package.

These assessments were conducted in a convenience sample of health facilities, which were located in purposively selected regions. Therefore, the findings might not be generalizable to the whole country or other countries in the region. Also, the facility coverage estimates reported in this document are not systematic so are not representative estimates of the true HepB-BD coverage in the five countries. However, they provide an additional data point with which to compare official national coverage estimates.

### Next steps/recommendations for introducing HepB-BD

A large amount of work remains to be done in the region to achieve the control goal. All five countries had considerable challenges in achieving high timely HepB-BD among both babies born in facilities and those born outside health facilities. While it is generally thought that vaccinating facility births is relatively straight forward and easy, these assessments show the missed opportunities and factors that contribute to low coverage among this cohort. They also demonstrate the enormous challenge countries will face in accessing babies born outside health facilities with timely HepB-BD. Countries seeking to introduce HepB-BD should offer universal vaccination to all newborns. Studies have found the targeted strategy to be ineffective^30^. If countries chose to implement targeted vaccination, they should develop systems to record maternal HBV status in delivery registers so babies can be tracked.

HCWs should be educated about the importance of timeliness of HepB-BD and provided with written guidelines and tools to augment their knowledge. Trainings should address knowledge gaps among HCWs and provide a consistent message: HepB-BD should be given ideally within 24 hours for maximum effectiveness, but if not possible, then it should still be given later. This difference should be distinguished in recording and reporting. Trainings should involve both public and private HCWs since both play an integral role in HepB-BD vaccination. Health facilities should develop SOPs to administer HepB-BD as soon after birth as possible, rather than on discharge, and offer vaccination services every day including weekends and public holidays. The JRF global standard indicator for HepB-BD is defined as doses given ≤24 hours after birth. However, JRF forms from the assessed countries did not have allowances to capture timing of HepB-BD. Countries should revise their recording and reporting tools to document timely birth doses, and any dose that is given before the first pentavalent vaccine. Reporting the two categories will help countries to account for all given doses. National delivery registers should also be modified to include dedicated columns for HepB with allocations for recording the date of administration.

Coordination between EPI and MCH is necessary to develop innovative strategies for reaching home births with timely HepB-BD. Babies who are born at home traditionally have lower vaccination coverage because they have less access to healthcare services. However, some births taking place outside health facilities might be attended to by skilled birth attendants (SBA), presenting an opportunity to vaccinate these babies^29^. HCWs also have a chance to counsel mothers on HepB-BD and importance of delivering in health facilities during ANC. This is a feasible strategy since it has been shown that >90% of pregnant women in 27 African countries attended at least one ANC visit, and 80–90% of pregnant women in an additional 11 countries had at least one ANC visit^22^. Assurance of timely HepB-BD can be an added impetus for women who have delivered at home to seek Post Natal Care. Many African countries have nomadic and semi-nomadic populations with a high proportion of home births that are not attended by SBAs. It is also difficult to estimate vaccine coverage in this population because of unclear denominators. Using community health workers to track pregnancies to deliver targeted messages and offer home-based PNC may improve timely HepB-BD and is a feasible approach.

Several strategies have been employed successfully in countries in the Western Pacific Region to increase HepB-BD administration to home births and can be implemented in Africa^22^,^31^. Use of vaccine out-of-cold chain (OCC) is a policy that might improve timely HepB-BD coverage in countries with low cold chain capacity and can support outreach vaccination services^31^. In October 2016, the WHO Strategic Advisory Group of Experts (SAGE) encouraged the re-labelling of monovalent HepB for OCC use and that countries should follow the Immunization Practices Advisory Committee recommendations for OCC^23^. Several countries indicated their interest in introducing HepB-BD in national EPI in the next few years during a Regional WHO consultation held in November 2016^15^. However, there is a need to conduct representative serological surveys to determine the true burden of chronic HBV and assess the impact of HepB vaccination. Findings from the sero-surveys can then be used evidence for national immunization technical advisory groups to approve HepB-BD introduction, thereby accelerating steps to achieve the regional goal. WHO has developed guidelines to support countries^12^,^18^,^32^. The renewed momentum to control viral hepatitis in Region is an opportunity for countries to invest in HepB-BD introduction which has been shown to be a cost-effective intervention^33^,^34^.

## Figures and Tables

**Table 1:** Facility characteristics and vaccine knowledge, practices, and management for the countries with Hepatitis B birth dose assessments^1^

	STP (n=5)	Nigeria (n=23)	Gambia (n=9)	Botswana (n=16)
*Background characteristics*				
*Total number of deliveries (median [range])*	336 (31–4,383)	278 (25–1,667)	1719 (500–6,000)	167 (19–6,000)
*100% of mothers stay >=24h post delivery*	5 (100%)	10 (43%)	3 (33%)	2 (13%)
*Staff Knowledge*				
*Received training on HepB-BD*	4 (80%)	14 (61%)	6 (56%)	2 (13%)
*Know that a mother can transmit HBV to her baby*	5 (100%)	23 (100%)	8 (92%)	12 (75%)
*Know that recommended HepB-BD administration is <24h of birth*	5 (100%)	23 (100%)	7 (71%)	14 (88%)
*Practices*				
*Vaccinate ALL newborns with HepB-BD*	0 (0%)	23 (100%)	9 (100%)	16 (100%)
*Follow standard written protocols for HepB-BD administration*	0 (0%)	6 (26%)	0 (100%)	0 (100%)
*Provide written documentation of HepB-BD to mother*	5 (100%)	22 (96%)	9 (100%)	16 (100%)
*Vaccinate in the delivery room*	5 (100%)	6 (26%)	0 (100%)	1 (6%)
*Administer to very low weight babies (<2kg)*	4 (80%)	9 (39%)	2 (22%)	0 (0%)
*Administer to premature babies*	3 (60%)	6 (26%)	1 (11%)	1 (6%)
*Administer to ill but stable babies*	1 (20%)	7 (30%)	2 (22%)	1 (6%)
*Provide HepB-BD outreach vaccination to home births*	0 (0%)	0 (0%)	0 (0%)	0 (0%)
*Charge patient for HepB-BD administration*	0 (0%)	2 (9%)	0 (0%)	2 (13%)
*Patients sometimes refuse HepB-BD*	0 (0%)	1 (4%)	0 (0%)	1 (6%)
*Offer HepB-BD vaccination daily*	5 (100%)	0 (0%)	0 (0%)	16 (100%)
*Require a physician order for HepB-BD*	0 (0%)	0 (0%)	0 (0%)	0 (0%)
*Vaccine management*				
*Stock out >2 weeks in 2014*	0 (0%)	0 (0%)	0 (0%)	2 (13%)
*Vaccine fridge is EPI-approved*	5 (100%)	12 (52%)	9 (100%)	16 (100%)
*Observed VVM stage 3-4 in fridge*	0 (0%)	1 (4%)	0 (0%)	0 (0%)
*Fridge monitored at least 2x/day*	5 (100%)	6 (26%)	9 (100%)	16 (100%)
*Vaccine obtained from MOH EPI*	5 (100%)	23 (100%)	9 (100%)	16 (100%)
*Implement multi-dose vial policy*		23 (100%)	9 (100%)	16 (100%)

1 A modified protocol was employed in Namibia as the HepB-BD assessment was embedded in a larger comprehensive EPI and surveillance review, and did not include comprehensive facility assessment component.

**Table 2:** Hepatitis B birth dose coverage, institutional births, and antenatal care visits in the 5 African countries with birth dose assessments

Country	% HBsAg prevalence (min %, max %)	Year HepB-BD introduced	Annual Births (1000s)^1^	Institutional deliveries %^2^	Births attended by SBA %^2^	>1 ANC visit %^2^
Botswana	5.3, 10.6 (35–37)	Pre 2000	55	100	95	94
Gambia	8.5, 9.1 (38–40)	1990	83	63	57	86
Namibia	7.8, 13.6 (41–43)	2014	72	87	88	97
Nigeria	6.7, 17.2 (44–46)	2004	7,133	36	38	61
Sao Tome and Principe^3^	6.1, 10 (47)	2002	6	91	93	98

1 Annual birth data is derived from the WHO Immunization Monitoring System (updated May 2016) http://apps.who.int/immunization_monitoring/globalsummary.

2 Data derived from UNICEF (updated February 2016) www.data.unicef.org

3 Sao Tome and Principe does not offer the birth dose universally, but follow a selective policy where infants of mothers that test HBsAg are offered vaccine

**Table 3:** National level characteristics and policies for the 5 countries with birth dose assessments

	STP	Nigeria	Gambia	Botswana	Namibia
National plan focusing on prevention and control of viral hepatitis	No	No	No	No	No
Designated govt. unit responsible for carrying out viral hepatitis activities	No	Yes	No	No	No
National representative sero-survey data showing HBV burden	No	No	No	No	No
National clinical guidelines for managing viral hepatitis	No	No	No	No	No
National guidelines/policy related to HepB-BD vaccination	Yes	Yes	Yes	Yes	Yes
Have an upper limit for timely HepB-BD vaccination (≤24 hrs.)	No	Yes	No	No	No
EPI recording tools allow for capture of timely (≤24 hrs.) and total BD doses	No	No	Yes	No	No
HepB-BD integrated in newborn care policy	No	No	No	No	No
MCH data recording tools capture HepB-BD administration	No	No	No	Yes	No
Outreach programs to vaccinate home births within 24 hours	No	No	No	No	No
